# Protective Effect of Platelet-Rich Plasma on Cisplatin-Induced Nephrotoxicity in Adult Male Albino Rats: Histological and Immunohistochemical Study

**DOI:** 10.1007/s12011-023-03742-9

**Published:** 2023-07-07

**Authors:** Melad N. Kelada, Amany Elagawany, Nancy Mohamed El Sekily, Mona El Mallah, Maha W. Abou Nazel

**Affiliations:** 1 Anatomy and Embryology department, Faculty of Medicine, University of Alexandria, Alexandria, Egypt; 2 Histology and Cell Biology Department, Faculty of Medicine, University of Alexandria, Alexandria, Egypt

**Keywords:** Cisplatin, Drug-induced nephrotoxicity, PRP, Morphometric parameters, Immunohistochemistry

## Abstract

Cisplatin is a potent antineoplastic drug that is used for treatment of many solid tumors. It has a wide range of adverse effects. Nephrotoxicity is the most common one of them. Platelet-rich plasma (PRP) is an autologous human plasma that activates the tissue regeneration through cell proliferation and differentiation. Study the role of PRP in amelioration of cisplatin-induced nephrotoxicity on the kidney of adult male albino rats by biochemical, morphometric, histological, and immunohistochemical studies. Thirty-five adult male albino rats were used. Thirty rats were included as experimental group and five were used to obtain the PRP. The experimental group was classified into as follows: control group which received 1mL of sterile saline by intraperitoneal injection (IP), cisplatin-treated group which received cisplatin 7.5 mg/kg IP in a single dose and cisplatin and PRP-treated group rats received cisplatin 7.5 mg/kg single IP dose followed by 1ml of PRP IP after 24 h of cisplatin injection. There was a significant increase in urea and creatinine levels in cisplatin-treated group in comparison to the control and the PRP groups. The kidneys of cisplatin-treated group showed distorted renal structure, where specimens of PRP-treated group revealed restoration of the classical appearance of the renal tissue similar to the control group. PRP has protective effects on renal structure and functions and it helps to ameliorate the histological changes induced by cisplatin.

## Introduction

Cisplatin (cis-diammine-dichloroplatinum) is a potent antineoplastic drug that is used for treatment of many solid tumors. It is used for treatment of lymphoma, stomach, esophagus, pancreas, bladder, head and neck, breast, lung, and testicular cancers. The curing rate of cisplatin in testicular cancer is very high and it is about 90% [1].

Cisplatin has a wide range of adverse effects that starts from mild ones up to severe toxicity in different organs. Nausea, vomiting, temporary hair loss, anemia, and dehydration are examples for mild adverse effects. Nephrotoxicity is the most common one of them. About 25–40% of the patients receiving cisplatin have impaired renal functions [1, 2].

Cisplatin nephrotoxicity is a dose limiting adverse effect; thus, different doses of cisplatin lead to different degrees of nephrotoxicity with different degrees of histopathological change. This nephrotoxicity may be manifested as acute kidney injury (AKI), chronic kidney injury (CKI), or any other clinical renal manifestations [3, 4].

Acute kidney injury (AKI)occurs in about 25–40% of cases treated with cisplatin, but chronic renal injury of high grades 3 or 4 is estimated to be up to 8.5%. The main difference between acute and chronic renal injury is the rate and duration in which decline of renal functions occurs [3, 4].

Platelet-rich plasma (PRP) is an autologous human plasma. It is a mixture of highly concentrated platelets, growth factors, and bioactive molecules. It is prepared by centrifugation of the patient whole blood at different speeds. It is known that normal platelet count is about 150,000–350,000/μL. Improvement in the soft tissue healing occurs in by platelet count up to 1000, 000/μL. This high count of platelets is found only in platelet-rich plasma (PRP) preparations. PRP could contain up to five times growth factors compared to normal plasma [5].

Platelet-rich plasma nowadays is proved to be used to treat nephrotoxicity induced by different agents because of its growth factors content. It activates the tissue regeneration through cell proliferation and differentiation. Epidermal growth factor (EGF) in addition to hepatocyte growth factor (HGF) was proved to enhance renal tubule cell regeneration and repair and accelerates the recovery of renal functions [6–8].

In this work, we study the role of platelet-rich plasma (PRP) in the amelioration of the cisplatin-induced nephrotoxicity in the kidney of adult male albino rats by biochemical morphometric, histological, and immunohistochemical studies.

## Materials and Methods

The present study was carried out on 35 male albino Wistar rats obtained from the Animal House of Faculty of Agriculture, Alexandria University.

Thirty rats aged about 8 weeks with average weight 225 ± 25 g were used as the experimental group. The other five rats were used as a source for the PRP.

The study protocol was approved by Ethics Committee of Faculty of Medicine, Alexandria University (IRB No: 00012098- FWA No: 00018699). Serial number 0106475 and following the guidelines for care and use of animals and in accordance and adherence with ARRIVE guidelines.

Rats were housed in a room temperature maintained at 24 degrees (24°C) on a 12:12-h light:dark cycle. Diet was administrated following the Egyptian Institute of Nutrition (EIN) recommendations. Diet was purchased from Tanta Oil and Soap Company (EL-Mahalla Al-Kubra Sector), Egypt. Diet components are as follows: Bran - cotton seed meal - yellow corn - molasses - limestone powder - table salt.

The animals were given food and water ad libitum.

The 30 adult male albino rats were randomly assigned to three groups:

Control group: ten rats received sterile saline 0.9% (1mL, single dose) by intraperitoneal injection on day one as a placebo.

Cisplatin-treated group: ten rats each received cisplatin (7.5 mg/kg, single dose) by intraperitoneal injection on day one.

Cisplatin and PRP-treated group: included ten rats each received cisplatin (7.5 mg/kg, single dose) by intraperitoneal injection on day on and received platelet rich plasma (1 mL, single dose) by intraperitoneal injection after injection of cisplatin by 24 h.

All experimental rats were left 14 days then sacrificed by cervical dislocation.

### Preparing Platelet-Rich Plasma [9]

The preparation was done under strict sterile conditions at Biochemistry Department, Faculty of Medicine, Alexandria University.

PRP was obtained from five rats aged about 8 weeks and with an average weight 300 ± 50 g. The whole blood of rats was drawn through cardiac puncture and transferred into test tubes including 3.2% sodium citrate. The blood was centrifuged at 400 g for 10 min and supernatant was transferred to another sterile tube, centrifuged again at 800 g for 10 min.

The top 2/3, which consists of platelet-poor plasma (PPP), was removed and discarded. The remaining layer (1/3) was considered as PRP and preserved in sterile ebindurf and frozen at −20°C.

#### Platelet Count

An automated cell counter was used to detect the PRP platelets count; the average was 2380×10^3^ platelets/μL which was 5 times the whole blood levels.

All experimental animals were subjected to the following studies:

I. Biochemical study:

Plasma urea and creatinine levels were measured on days 1 and 14. The blood samples (2ml for each sample) were collected from retro orbital venous plexus in a dry clean non-heparinized test tube for assessing the serum level of urea and creatinine. Using urea (rat) ELISA kit and creatinine (rat) ELISA kit, Abbot, Austria, steps were done following the manufacturer’s instructions.

II. Histological study:

A. H&E stain [10]

The kidney was dissected, cut into 5-mm pieces, and immediately fixed in 10% formol saline for 72 h. Paraffin blocks were made and sectioned into 4-μm-thick sections.

The sections were stained with H&E and studied using a light microscope (Olympus CX41 Binocular LED–Sample Microscope) at Center of Excellence for Research in Regenerative Medicine and its Applications (CERRMA), Faculty of Medicine.

B. Masson’s trichrome stain

Masson’s trichrome stain was used to detect fibrosis in the renal tissue by staining collagen with blue color [11].

III. Periodic-acid Schiff’s (PAS) stain

This stain is a histochemical technique used to detect neutral polysaccharides which are present in the basement membrane of the renal glomerulus and renal tubules giving a magenta-red color [11].

IV. Immunohistochemical study

Sections from paraffin blocks were immunohistochemically studied using streptavidin-biotin immune-enzymatic antigen detection system. The monoclonal antibody was anti caspase-3 kit for the detection of apoptotic cells in renal tissue using imaging analysis system [12].

III. Morphometric study:

After histological staining of the rat kidneys, digital images were taken under the objective lens using Olympus CX41 Binocular LED – Sample Microscope at (CERRMA). Morphometric analysis was carried on using computerized image analysis system Image J. [13].

The following morphometric parameters were measured:

1. The percentage of abnormal tubules (proximal and distal) in relation to the total tubules (tubular injury)

It was measured semi-quantitatively using Image J. About 20 cortical fields (×400 magnification) of periodic acid Schiff (PAS)-stained sections were examined [14].

Tubular injury was defined as tubular dilation, tubular atrophy and tubular cast formation, sloughing of tubular epithelial cells, vaculation of the epithelial cells or loss of the brush border, and thickening of the tubular basement membrane [15].

The following scoring system was used: score 0, no tubular injury; score 1, <10% of tubules injured; score 2, 10–25% of tubules injured; score 3, 25–50% of tubules injured; score 4, 50–74% of tubules injured; score 5, >75% of tubules injured [14].

2. The percentage of fibrosis [16]

The percentage of fibrosis was performed by measuring the presence of interstitial fibrosis in Masson’s trichrome-stained sections from each kidney. Digital images of at least 20 cortical fields (× 400 magnification) were examined and the percentage of fibrosis was measured using Image J program.

The following scoring system was used: score 0, no evidence of interstitial fibrosis; score 1, <25% involvement; score 2, 25 to 50% involvement; score 3, >50% involvement.

3. The percentage of apoptosis [17]

The percentage of apoptosis was performed by measuring the percentage of anti-caspase 3 positive areas in anti-caspase 3-stained sections from each kidney using Image J program.

4. The corpuscular parameters (the perimeter of the renal corpuscle (μm), the Feret’s diameter of the renal corpuscle (μm), the percentage of the surface area of Bowman’s space in relation to the surface area of the renal corpuscle, the circularity of the renal corpuscle)

The corpuscular parameters were measured in about 5 fields (× 400 magnification) of H&E-stained sections using Image J program. Only renal corpuscle with clearly demarcated vascular and tubular poles was included in our study [18].

The perimeter is the length of the outline of the renal corpuscle. Feret’s diameter is the longest distance between any two points along the boundary of the renal corpuscle [13].

The surface area of the renal corpuscle and the Bowman’s space were measured in the green channel of digital colored RGB (red-green-blue) images because the main relevant part of the renal corpuscle information appears in the green channel. Then, the image was binarized as the Image J program for particles analysis is used to analyze objects on binarized images only [19].

The circularity of the renal corpuscle was determined by the following formula: (4π* surface area) / (perimeter^2^), with a value of 1.0 indicating a perfect circle [13].

5. The glomerular basement membrane thickness (μm)

The glomerular basement membrane was measured in about 5 fields of PAS-stained sections from each kidney. It was estimated as a mean distance after manual tracing of two lines along both sides of it [13].

### Statistical Analysis

Morphometric data was analyzed with SPSS software package version 20.0.

Quantitative data were described using mean and standard deviation for normally distributed data. Qualitative data were described using number and percent. Comparison between different groups regarding categorical variables was tested using chi-square test. For normally distributed data, comparison between more than two populations was analyzed by *F*-test (ANOVA). Significance test results were quoted as two-tailed probabilities. Significance of the obtained results was judged at the 5% level [15].

## Results

### Biochemical Findings

There was a significant difference in the plasma creatinine level of cisplatin-treated group before and after administration of the drug with a very high level of it after administration of the drug (Table 1).

**Table 1:** Comparison between the three studied groups according to the change in plasma creatinine level and change in urea level between the 14th day and 1st day of the experiment

	Control (*n* = 10)	Cisplatin treated (*n* = 10)	PRP treated (*n* = 10)	*H*	*p*
Change in plasma creatinine level					
Min.–Max.	−0.21–0.10	0.16–2.20	−0.15–0.20	19.665^*^	<0.001^*^
Mean ± SD.	−0.04 ± 0.12	1.17 ± 0.59	0.03 ± 0.11		
Median	−0.04	1.16	0.0		
*p*_1_		<0.001^*^	0.358		
*p*_2_			0.001^*^		
Change in urea level					
Min.–Max.	−3.0–5.0	32.0–107.0	−12.0–32.0	21.422^*^	<0.001^*^
Mean ± SD.	0.80 ± 2.97	71.40 ± 22.17	13.60 ± 13.80		
Median	1.0	72.0	16.50		
*p*_1_		<0.001^*^	0.137		
*p*_2_			0.002^*^		

*SD*, standard deviation

*H*, *H* for Kruskal-Wallis test, pairwise comparison bet. each 2 groups was done using post hoc test (Dunn’s for multiple comparisons test)

*p*, *p* value for comparing between the three studied groups

*p*_*1*_, *p* value for comparing between control and each other groups

*p*_*2*_, *p* value for comparing between cisplatin-treated and PRP-treated

^*^Statistically significant at *p* ≤ 0.05

No significant difference in change in plasma urea level between control group and cisplatin and PRP-treated group. Change in the plasma urea level was significantly higher in cisplatin-treated group than in control group. Change in the plasma urea level was significantly higher in cisplatin-treated group than in cisplatin and PRP-treated group (Table 1).

### Histological Results

#### H&E Stain

##### Control Group (Fig. 1)

**Fig. 1:**
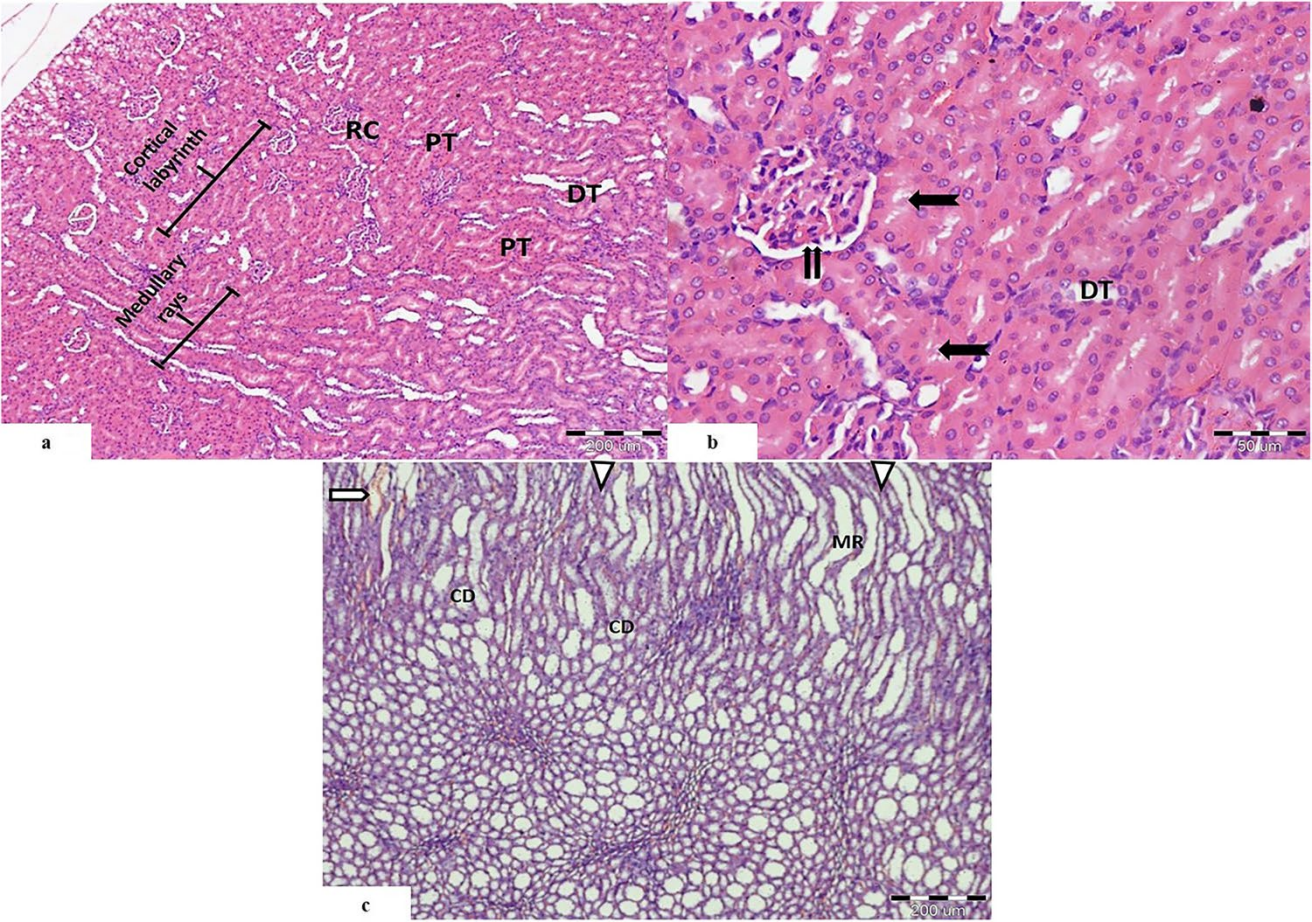
Light photomicrographs of rat’s kidney control group showing **a** renal cortex arranged into cortical labyrinths and medullary rays. It shows normal appearance of the renal corpuscle (RC), proximal convoluted tubules (PT), and distal convoluted tubules (DT). **b** Higher magnification showing the Bowman’s space of the renal corpuscle (  ). Proximal convoluted tubules showing prominent brush border (  ). The distal convoluted tubules (DT) appear with wider lumen and cuboidal cells with rounded nuclei. **c** The medulla is seen occupied by medullary rays (MR), collecting ducts (CD), renal interstitium (  ), and blood vessels (  ). (H&E. Mic.Mag **a** ×100, **b** ×400, and **c** ×100)

On low magnification, specimens of rats’ kidneys appeared with classical structure, divided into as follows: outer cortex and inner medulla. The cortex was occupied by nephrons. The nephron is formed from renal corpuscles and tubules. The cortex was organized into cortical labyrinths containing the corpuscles, proximal, and distal convoluted tubules and between them the medullary rays containing straight tubules and cortical collecting ducts (Fig. 1a)

High magnification revealed normal appearance of the renal corpuscle showing normal Bowman’s space and normal glomerular capillaries with urinary and vascular pole. The proximal convoluted tubules showed pyramidal cells deeply eosinophilic with rounded basal nuclei and prominent brush border. Distal convoluted tubules appeared with wider lumen and cuboidal cells with rounded nuclei. The distal convoluted cells become crowded close to the vascular pole of the renal corpuscle to form the macula densa (Fig. 1b).

The renal medulla showed parallel medullary rays which were formed from the straight part of proximal and distal tubules in addition to the thin part of loop of Henle. It is formed also from collecting ducts which appears as circle profiles. In between the tubules, there was the renal interstitium (Fig. 1c).

##### Cisplatin-Treated Group (Fig. 2)

**Fig. 2:**
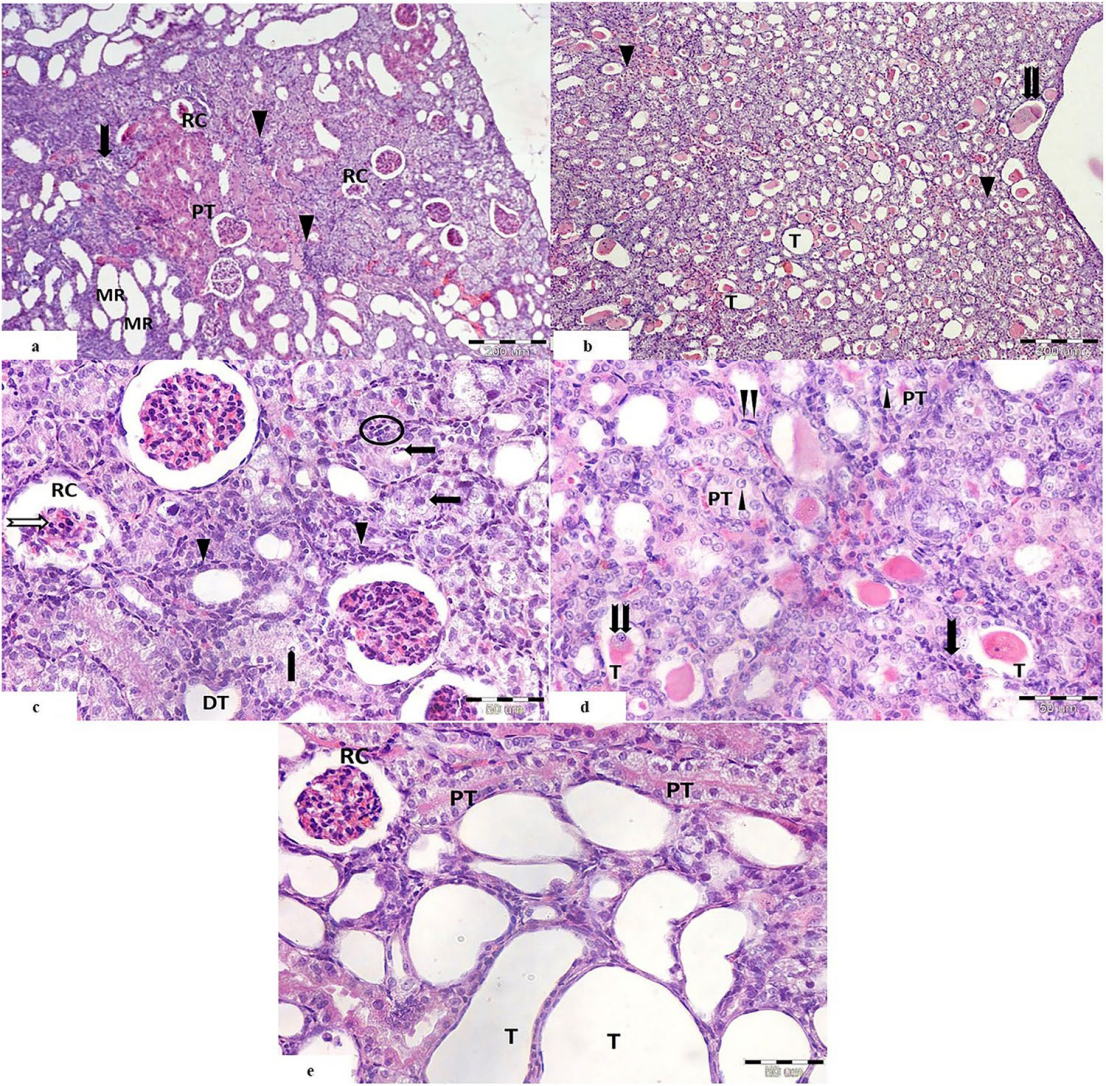
Light photomicrographs of a section of renal cortex from cisplatin-treated group showing **a** the cortex with distorted renal corpuscles (RC), ballooning of the tubules of the medullary rays (MR), intense cellular infiltration (  ) among the degenerated tubules (  ), and focal areas of proximal tubules with acidophilic cytoplasm (PT). **b** The medulla shows distorted tubules with multiple hyaline casts within their lumina (  ). Some of the tubules appears dilated (T), and others show obliterated lumen (  ). **c** The renal corpuscles (RC) appear with shrunken glomerular tuft and pyknotic nuclei (  ). Peritubular cellular infiltration is noticed around the distorted tubules (  ). The proximal convoluted tubules show vaculations (  ), bizzare shaped nuclei of the lining cells (  ), and extruded cells in the lumen with loss of brush border and basophilic cytoplasm (  ). Distorted distal convoluted tubules with flattened cells are seen (DT). **d** The proximal tubules (PT) show karyolitic nuclei (  ). Some tubules (T) show hyaline casts and exfoliated cells within their lumina (  ). There are peritubular cellular infiltration (  ) and proliferating interstitial fibroblasts (  ). **e** Renal corpuscle (RC) appears with shrunken glomerular tuft and dark nuclei. Many tubules show severe ballooning (T). Cells of proximal convoluted tubules show vaculations with loss of brush boarder (PT). (H&E. Mic.Mag **a**, **b** ×100, **c**–**e** ×400)

On low magnification, cisplatin-treated group showed abnormal renal corpuscles with shrunken glomerular capillaries and widened Bowman’s space. Intense cellular infiltration was seen among degenerated tubules which appeared basophilic. Focal areas of normally appearing proximal convoluted tubules with acidophilic cytoplasm were seen among the degenerated tubules. Tubules of the medullary rays showed severe ballooning (Fig. 2a).

The renal medulla of the same group showed distorted tubules with multiple hyaline casts filling their lumen. Some of them were dilated and others showed obliterated lumina (Fig. 2b).

On high magnification, there was massive affection of the proximal convoluted tubules which appeared with extensive vaculations, basophilic cytoplasm, extruded cells in the lumen with loss of the brush borders and collapsed tubules. The nuclei of the lining cells appeared bizarre shaped. There was excessive peritubular cellular infiltration. Renal corpuscles with shrunken glomerular capillaries, widened Bowman’s space, extravasated RBCs, and pyknotic nuclei were also seen (Fig. 2c).

Distal convoluted tubules appeared distorted with flattened cells, and others were extensively dilated. Congested blood vessels with extravasation of the red blood corpuscles surrounded by cellular infiltration and also around obliterated tubules were observed (Fig. 2c).

Some renal tubules showed eosinophilic hyaline casts and exfoliated cells in their lumina. The nuclei of the lining cells of the proximal convoluted tubules showed irregularities with karyolysis. Many proliferating interstitial fibroblasts were also depicted (Figs. 2d, 2e).

##### Cisplatin and PRP-Treated Group

**Fig. 3:**
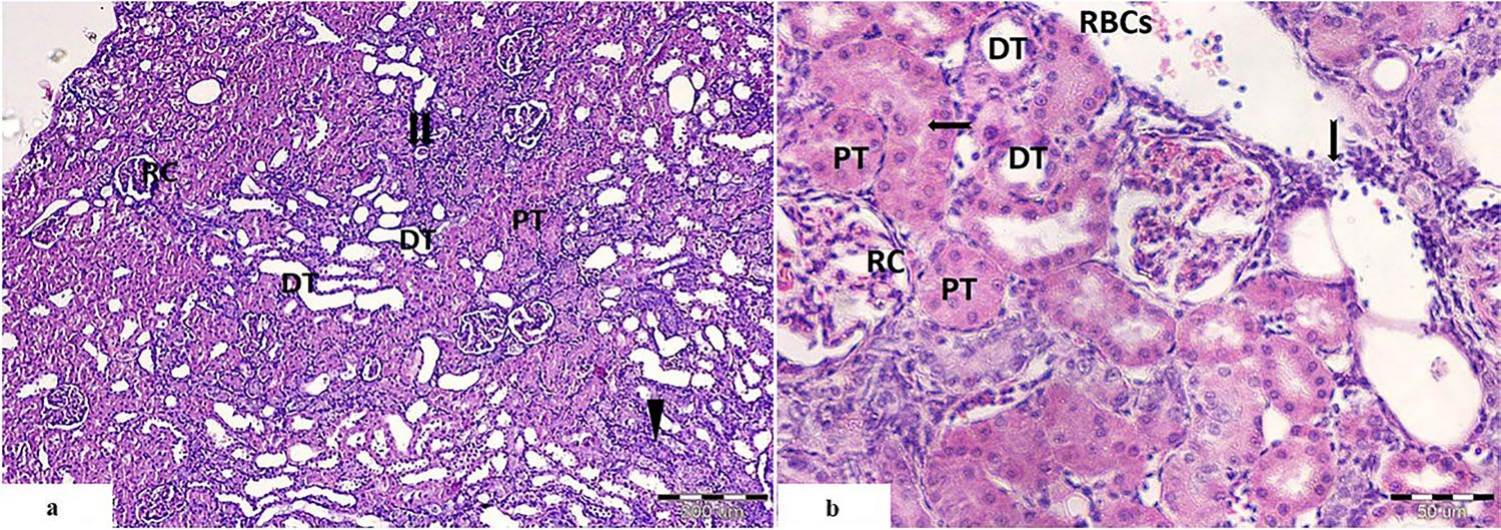
Light photomicrographs of rat’s kidney from cisplatin and PRP-treated group showing **a**, **b** the renal corpuscles appear nearly normal with bifurcated glomerular tuft (RC), many normal proximal convoluted tubules with eosinophilic cytoplasm and intact epithelial lining (PT). Few tubules are obliterated, with basophilic cytoplasm (  ), and others appeared degenerated with hyalinized material in their lumen (  ). **b** Distal convoluted tubules appear with cuboidal epithelial lining (DT). Widened interstitial space with evident cellular infiltration (  ) and extravasated RBCs (RBCs) are also seen (  ); brush border of PCT. (H&E. Mic.Mag **a** ×100 and **b** ×400)

On low magnification, the cortex showed nearly normal renal corpuscles showing bifurcated glomeruli. Most of the proximal tubules appeared normal with eosinophilic cytoplasm and intact epithelial lining, but few of them appeared with basophilic cytoplasm, obliterated, or degenerated with hyaline material in the lumen. Distal tubules were dilated but less than that of the cisplatin-treated group (Fig. 3a).

On high magnification, the cortex showed restored structure of the proximal convoluted tubules which appeared eosinophilic with intact epithelial lining and intact brush border. Distal convoluted tubules appeared with cuboidal epithelial lining. Widened interstitial spaces with evident cellular infiltration and extravasated RBCs were also seen. The renal corpuscles were nearly normal with normal Bowman’s space and bifurcated glomerular capillary (Fig. 3b).

#### Masson’s Trichrome Stain

##### Control Group

**Fig. 4:**
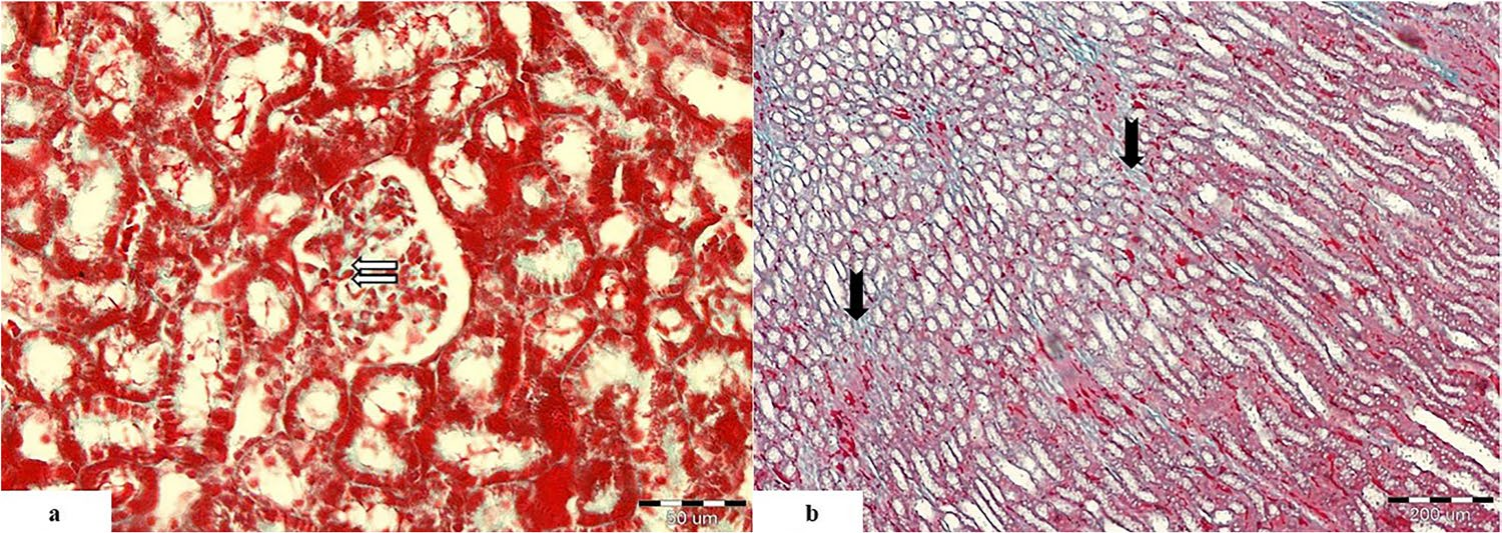
Light photomicrographs of the renal cortex of the control group showing **a** normal distribution of the collagen fibers in the mesangium of the renal corpuscle (  ). **b** The medulla shows some collagen fibers in the peritubular interstitium (  ). (Massonʼs trichrome. Mic.Mag **a** ×400 and **b** ×100)

The renal cortex showed normal structure with normal distribution of the collagen fibers in the mesangium of the renal corpuscle (Fig. 4a).The renal medulla showed some collagen fibers in the peritubular interstitium which were stained blue (Fig. 4b).

##### Cisplatin-Treated Group

**Fig. 5:**
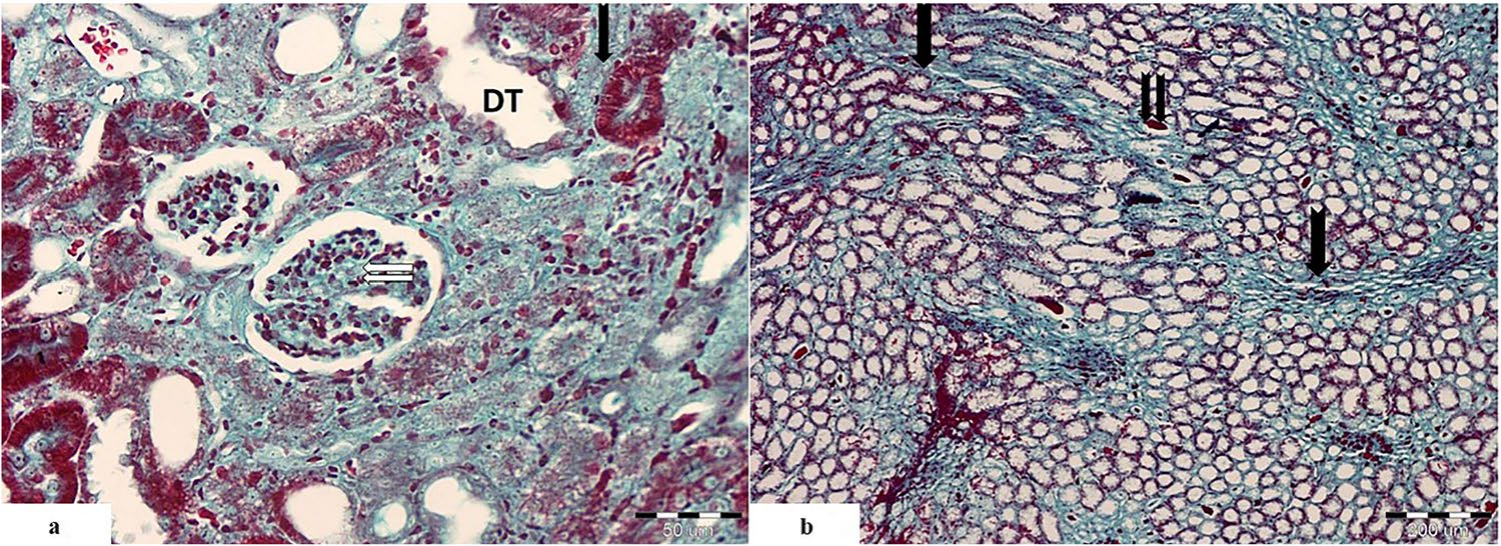
Light photomicrographs of renal cortex from the cisplatin-treated group showing **a** intense collagen fiber deposition in the cortical peritubular interstitium (  ) and in the mesangium of the renal corpuscle (  ). Some distal tubules show dilatation (DT). **b** Intense collagen fiber deposition in the medullary interstitium (  ). Some tubules show hyaline casts deposition (  ). (Massonʼs trichrome. Mic.Mag **a** ×400 and **b** ×100)

The renal cortex showed intense collagen fiber deposition in the interstitium around the tubules in addition to the mesangium of the renal corpuscle (Fig. 5a).

The renal medulla showed intense collagen fiber deposition in the medullary interstitium in addition to multiple hyaline casts in the lumen of many tubules (Fig. 5b).

##### Cisplatin and PRP-Treated Group

**Fig. 6:**
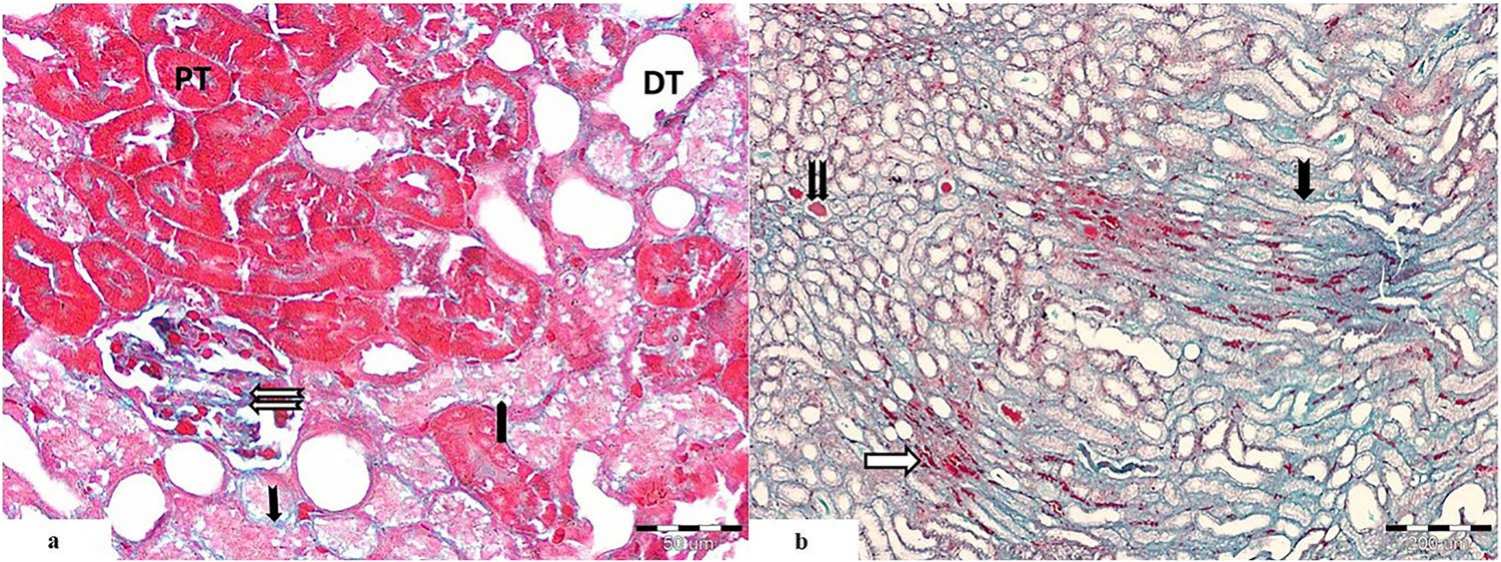
Light photomicrographs of renal cortex from cisplatin and PRP-treated group showing **a** mild collagen fibers deposition in the mesangium of the renal corpuscles (  ) and peritubular interstitium (  ). Some proximal tubules are normal (PT), whereas others are abnormal with prominent vaculations (  ). Some of the distal tubules are dilated (DT). **b** Moderate collagen fiber deposition in the medullary interstitium (  ). Some tubules show hyaline cast deposition (  ). There are vasa recta and extravasated RBCs in between the parallel tubules (  ). (Masson trichrome. Mic.Mag **a** ×400 and **b** ×100)

The renal cortex showed mild to moderate collagen fibers deposition in the interstitium around the tubules in addition to the mesangium of the renal corpuscle (Fig. 6a).

The renal medulla showed moderate collagen fibers deposition in the medullary interstitium in addition to multiple hyaline casts in the lumen of some tubules. There were also evident vasa recta in between the parallel tubules (Fig. 6b).

Comparison between the studied groups as regards percentage of fibrosis showed the following (Table 2):

- Control group: the renal tissue showed low percentage of fibrosis when compared to other studied groups. The mean value of percentage of fibrosis was about 7.88% (score 1).
- Cisplatin-treated group: the renal tissue showed the highest percentage of fibrosis when compared to other studied groups. The mean value of percentage of fibrosis was about 51.42% (score 3).
- Cisplatin and PRP-treated group: showed low percentage of fibrosis when compared to cisplatin-treated group. The mean value of percentage of fibrosis was about 18.07% (score 1).

**Table 2:** Comparison between the three studied groups according to percentage of fibrosis

Percentage of fibrosis	Control (*n* = 5)	Cisplatin treated (*n* = 5)	PRP treated (*n* = 5)	*F*	*p*
Min.–Max.	5.75–13.22	48.87–54.06	10.18–25.07	186.977^*^	<0.001^*^
Mean ± SD.	7.88 ± 3.08	51.42 ± 1.98	18.07 ± 5.31		
Median	7.23	50.85	18.50		
***p***_**1**_		<0.001^*^	0.003^*^		
***p***_**2**_			<0.001^*^		

*SD*, standard deviation

*F*, *F* for one-way ANOVA test, pairwise comparison bet. each 2 groups was done using post hoc test (Tukey)

*p*, *p* value for comparing between the three studied groups

*p*_*1*_, *p* value for comparing between control and each other groups

*p*_*2*_, *p* value for comparing between cisplatin treated and PRP treated

^*^Statistically significant at *p* ≤ 0.05

There was a significant decrease in the percentage of fibrosis in the renal tissue in cisplatin and PRP-treated group in comparison with cisplatin-treated group.

#### Periodic Acid Schiff Stain

##### Control Group

**Fig. 7:**
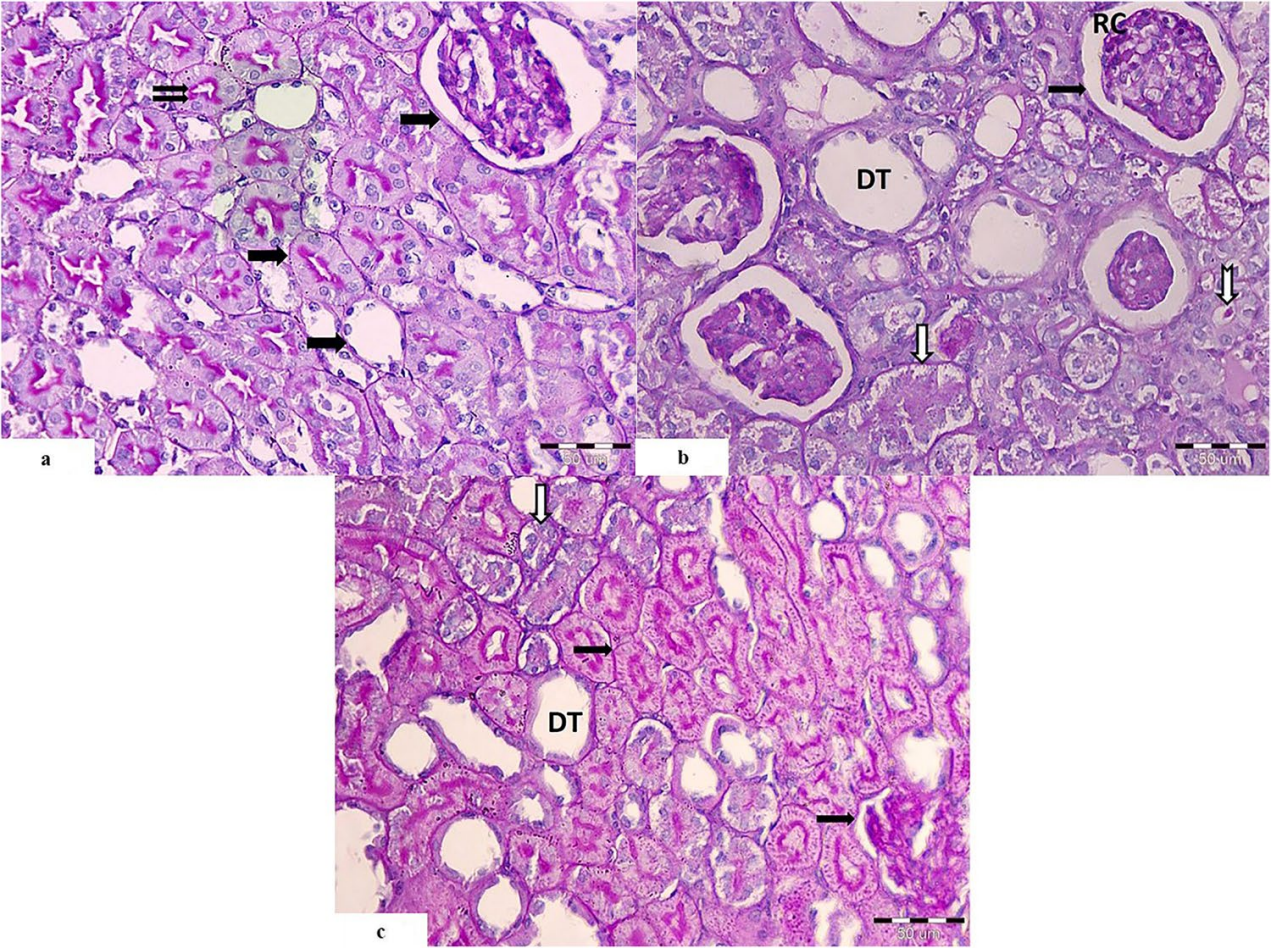
Light photomicrographs of renal cortex revealing **a** control group illustrates positive PAS reaction in the glomerulus, the basement membrane of the renal tubules (  ), and brush border of proximal convoluted tubules (  ). It is stained magenta red with the PAS stain. **b** The cisplatin-treated group shows thickened basement membrane of the glomerular tuft (  ). One of the glomeruli shows hyper cellularity (RC). Many proximal convoluted tubules appear degenerated with thickened basement membrane around them (  ). Some tubules show hyaline casts within its lumen (  ). Some distal tubules show dilatation (DT). **c** Cisplatin and PRP-treated group shows positive reaction within the basement membrane of the renal corpuscle and the proximal convoluted tubules (  ). Few of them appear degenerated with thickened basement membrane (  ). Some distal tubules show dilatation with thickened epithelial basement membrane (DT), and others appear nearly normal (PAS. Mic.Mag ×400)

The renal cortex showed positive PAS reaction with normal thickness of basement membrane of the glomeruli and the proximal and distal convoluted tubules. The basement membrane appears intact, and the brush border of the proximal convoluted tubules showed positive reaction. The basement membrane and the brush border were stained magenta red with the periodic acid Schiff stain (Fig. 7a).

##### Cisplatin-Treated Group

Strong positive reaction of PAS in the basement membrane of the glomeruli, proximal convoluted tubules, and distal convoluted tubules, while the brush border of the proximal convoluted tubules showed weak reaction. The proximal tubules showed thickened basement membrane around degenerated proximal tubules. Some of them appeared with disturbed basement membrane and many of them showed disrupted brush border. Many distal tubules appeared dilated with thickened basement membrane. Some tubules showed hyaline casts (Fig. 7b).

##### Cisplatin and PRP-Treated Group

Positive reaction of PAS in the basement membrane of the glomeruli, proximal convoluted tubules, distal convoluted tubules, and in the brush border of the proximal convoluted tubules. Nearly normal thickness of the basement membrane around both the renal tubules and glomerulus was depicted. Some proximal tubules appeared degenerated with thickened epithelial basement membrane and disrupted brush border, and others appeared with intact basement membrane and brush border. Some distal tubules appeared dilated with thickened basement membrane, and others appeared nearly normal (Fig. 7c).

#### Immunohistochemical Findings

Immunohistochemical study on paraffin sections of rats’ kidneys was conducted by staining with monoclonal antibody caspase-3 for detection of active caspase-3 in apoptotic cells. The immunohistochemical staining appeared as intracytoplasmic ± nuclear brown coloration of the renal tubules in addition to the renal interstitium.

##### Control Group

**Fig. 8:**
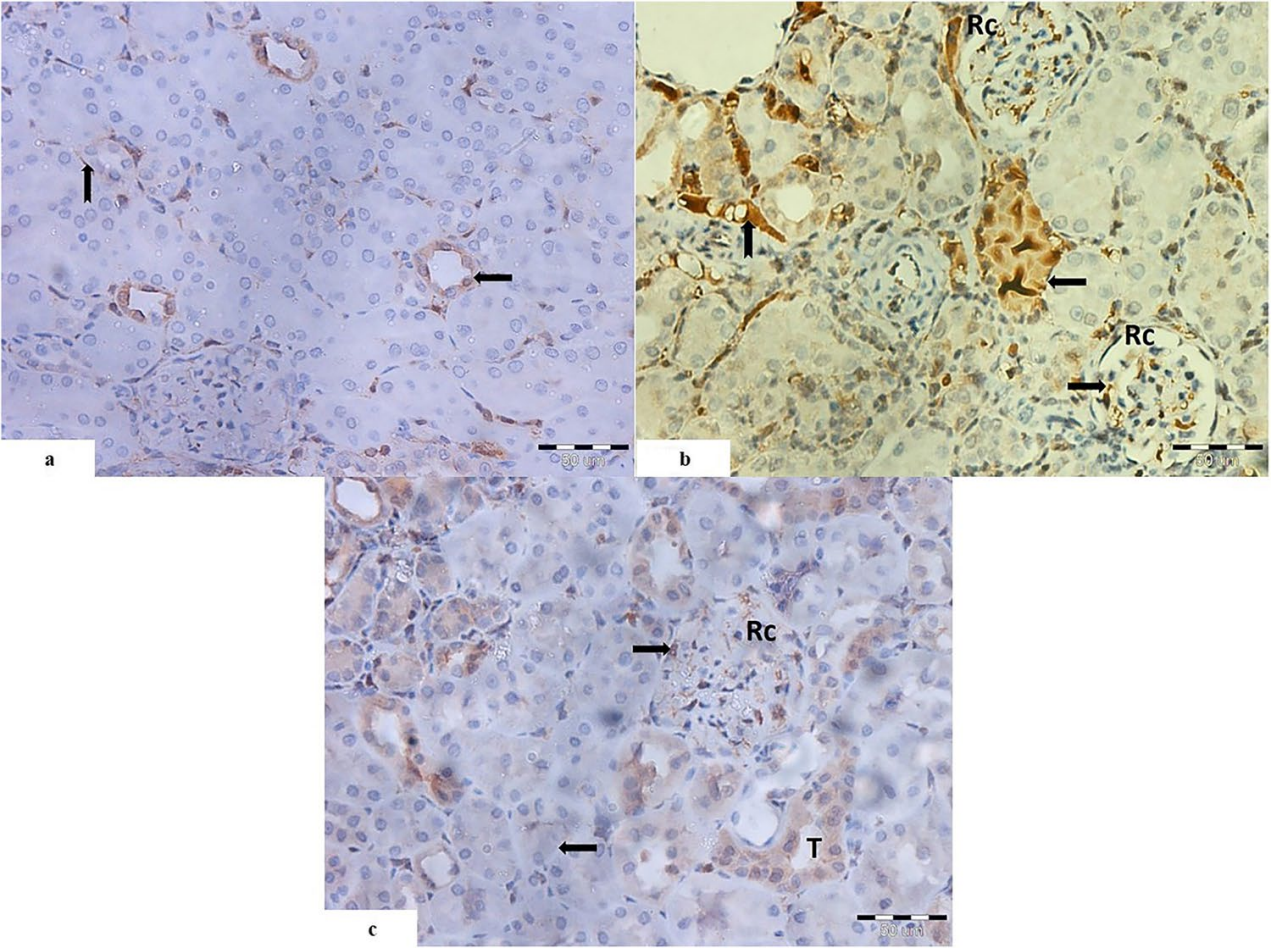
Light photomicrographs of section of the renal cortex: **a** the control group shows weak immunohistochemical reaction within the cells of the distal renal tubules (  ) and the interstitium (  ). **b** Cisplatin-treated group shows intense positive reaction in the degenerated tubules with obliterated lumen (  ). The interstitium shows strong positive reaction (  ). Also, there is strong positive reaction within the cells of the glomerular capillary tuft (  ) of the renal corpuscle (RC). **c** Cisplatin and PRP-treated group shows positive reaction within the lining cells of some tubules (T), and others show weak reaction (  ). Few cells of the glomerular tuft show positive reaction (  ) (caspase-3 immunohistochemical staining. Mic.Mag ×400)

There was a weak reaction within the cells of the renal tubules in addition to the interstitial fibroblasts (Fig. 8a).

##### Cisplatin-Treated Group

The renal cortex showed intense positive reaction especially in the degenerated tubules with obliterated lumen, the renal interstitium, and among the cells of the glomerular capillary tuft. Some tubules showed extruded degenerated cells within their lumina (Fig. 8b).

##### Cisplatin and PRP-Treated Group

The renal cortex showed positive reaction in some renal tubules, and others showed very weak reaction. There was weak positive reaction within few cells of the glomerular capillary tuft (Fig. 8c)

Comparison between the studied groups as regards presence of active caspase 3 in apoptotic cells (intensity, density) showed the following (Table 3):

- Control group: The renal tissue of the control group showed low caspase 3 positivity in apoptotic cells when compared to other studied groups, with mild intensity and mean staining density 2.49% (score 1).
- Cisplatin-treated group: The renal tissue of the cisplatin-treated group showed the highest degree of caspase 3 positivity in apoptotic cells when compared to other studied groups. The group showed severe intensity with mean staining density 29.04% (score 2).
- Cisplatin and PRP-treated group: The renal tissue of the PRP-treated group showed low caspase 3 positivity in apoptotic cells when compared to other studied groups, with a mild intensity and mean staining density 8.95% (score 1).

**Table 3:** Comparison three studied between the groups according to percentage of positive anti-caspase 3 reaction

Percentage of positive anti-caspase 3 reaction	Control (*n* = 5)	Cisplatin treated (*n* = 5)	PRP treated (*n* = 5)	*F*	*p*
Min.–Max.	2.04–3.17	25.10–32.76	7.70–9.70	319.551^*^	<0.001^*^
Mean ± SD.	2.49 ± 0.48	29.04 ± 2.84	8.95 ± 0.85		
Median	2.43	29.17	9.20		
***p***_**1**_		<0.001^*^	<0.001^*^		
***p***_**2**_			<0.001^*^		

*SD*, standard deviation

*F*, *F* for one-way ANOVA test, Pairwise comparison bet. each 2 groups was done using post hoc test (Tukey)

*p*, *p* value for comparing between the three studied groups

*p*_*1*_, *p* value for comparing between control and each other group

*p*_*2*_, *p* value for comparing between cisplatin treated and PRP treated

^*^Statistically significant at *p* ≤ 0.05

### Morphometric Study

Cisplatin-treated group showed a wide range of morphological damage. The tubules were considered abnormal when one of the following histological changes was found: severe vaculations of the cells of the proximal tubules, pyknotic changes in the nuclei, loss of brush border, severe ballooning of the distal convoluted tubules with attenuated lining cells, hyaline casts and extruded cells within the lumen of the tubules, increase thickness of the tubular and glomerular basement membrane. Shrunken or dilated renal corpuscles with dilatation of the Bowman space were also scored.

1. Comparison between the three studied groups according to percentage of normal and abnormal tubules showed the following (Table 4):

- Control group showed nearly normal tubules. The mean value of the percentage of abnormal/total tubules was 6.66% (score 1).
- Cisplatin-treated group showed abnormal tubules within nearly the total field. The mean value of the percentage of abnormal/total tubules was 91.11% (score 5).
- Cisplatin and PRP-treated group showed increase in the number of normal tubules. The mean value of the percentage of abnormal/total tubules was 32.10% (score 3).
  2. Comparison between the three studied groups according to the Feret’s diameter (μm) of the renal corpuscle showed the following (Table 5):

**Table 4:** Comparison between the three studied groups according to percentage of normal and abnormal tubules

Percentage tubules	Control (*n* = 10)	Cisplatin treated (*n* = 10)	PRP treated (*n* = 10)	*H*	*p*
Normal/total					
Min.–Max.	86.27–98.04	3.45–17.19	56.76–78.33	25.841^*^	<0.001^*^
Mean ± SD.	93.34 ± 4.32	8.89 ± 4.88	67.90 ± 9.33		
Median	94.15	7.93	68.61		
*p*_1_		<0.001^*^	0.011^*^		
*p*_2_			0.011^*^		
Abnormal/total					
Min.–Max.	1.96–13.73	82.81–96.55	21.67–43.24	25.841^*^	<0.001^*^
Mean ± SD.	6.66 ± 4.32	91.11 ± 4.88	32.10 ± 9.33		
Median	5.85	92.07	31.39		
*p*_1_		<0.001^*^	0.011^*^		
*p*_2_			0.011^*^		
Normal/abnormal					
Min.–Max.	628.6–5000.0	3.57–20.75	131.3–361.5	25.841^*^	<0.001^*^
Mean ± SD.	2302.5 ± 1685.9	10.05 ± 6.04	237.0 ± 98.25		
Median	1730.0	8.62	232.4		
*p*_1_		<0.001^*^	0.011^*^		
*p*_2_			0.011^*^		

*SD,* standard deviation

*H*, *H* for Kruskal-Wallis test, pairwise comparison bet. each 2 groups was done using post hoc test (Dunn’s for multiple comparisons test)

*p*, *p* value for comparing between the three studied groups

*p*_*1*_, *p* value for comparing between control and each other group

*p*_*2*_, *p* value for comparing between cisplatin treated and PRP treated

^*^Statistically significant at *p* ≤ 0.05

**Table 5:** Comparison between the three studied groups according to Feret’s diameter of the renal corpuscle, perimeter of the renal corpuscle, percentage of the surface area of Bowman’s space, circularity of the renal corpuscle, and basement membrane thickness in micrometer

	Control (*n* = 10)	Cisplatin treated (*n* = 10)	PRP treated (*n* = 10)	*F*	*p*
Feret’s diameter of the renal corpuscle					
Min.–Max.	105.58–130.23	130.71–191.26	110.66–150.13	20.712^*^	<0.001^*^
Mean ± SD.	117.49 ± 9.04	161.96 ± 23.79	126.15 ± 12.55		
Median	120.21	167.82	125.79		
*p*_1_		<0.001^*^	0.473		
*p*_2_			<0.001^*^		
Perimeter of the renal corpuscle					
Min.–Max.	296.9–308.4	313.3–397.3	298.2–304.8	42.493^*^	<0.001^*^
Mean ± SD.	302.1 ± 3.46	356.9 ± 26.18	302.4 ± 2.27		
Median	302.4	360.1	303.2		
*p*_1_		<0.001^*^	0.999		
*p*_2_			<0.001^*^		
Percentage of the surface area of Bowman’s space					
Min.–Max.	13.09–21.38	28.11–77.19	13.09–21.38	42.248^*^	<0.001^*^
Mean ± SD.	15.89 ± 2.55	45.94 ± 14.35	15.31 ± 2.38		
Median	14.64	43.73	14.59		
*p*_1_		<0.001^*^	0.987		
*p*_2_			<0.001^*^		
Circularity of the renal corpuscle					
Min.–max.	0.967–0.990	0.877–0.910	0.959–0.975	308.106^*^	<0.001^*^
Mean ± SD.	0.973 ± 0.007	0.896 ± 0.010	0.967 ± 0.006		
Median	0.971	0.900	0.969		
*p*_1_		<0.001^*^	0.275		
*p*_2_			<0.001^*^		
Basement membrane thickness in micrometer					
Min.–Max.	0.77–0.97	1.87–2.15	0.97–1.97	33.952^*^	<0.001^*^
Mean ± SD.	0.87 ± 0.09	2.03 ± 0.11	1.47 ± 0.36		
Median	0.87	2.05	1.44		
*p*_1_		<0.001^*^	0.003^*^		
*p*_2_			0.005^*^		

*SD*, standard deviation

*F*, *F* for one-way ANOVA test, pairwise comparison bet. each 2 groups was done using post hoc test (Tukey)

*p*, *p* value for comparing between the three studied groups

*p*_*1*_, *p* value for comparing between control and each other groups

*p*_2_, *p* value for comparing between cisplatin treated and PRP treated

^*^Statistically significant at *p* ≤ 0.05

The cisplatin-treated group showed significant increase in the mean value of Feret’s diameter of the corpuscle in comparison with the PRP-treated group and the control group.

3. Comparison between the three studied groups according to the perimeter (μm) of the renal corpuscle showed the following (Table 5):

There was significant increase in the perimeter of the renal corpuscle in the cisplatin-treated group in comparison with other groups.

4. Comparison between the three studied groups according to the percentage of the surface area of Bowman’s space in relation to the surface area of the corpuscle showed the following (Table 5):

There was significant increase in the percentage of the surface area of Bowman’s space in relation to the surface area of the corpuscle in the cisplatin-treated group in comparison with other groups.

5. Comparison between the three studied groups according to the circularity of the renal corpuscle showed the following (Table 5):

There was significant decrease in the circularity of the renal corpuscle in the cisplatin-treated group in comparison with other groups.

6. Comparison between the three studied groups according to the thickness of the basement membrane showed the following (Table 5):

There was significant increase in the basement membrane thickness of the cisplatin-treated group in comparison with other groups. Also, there was significant increase in the basement membrane thickness of the cisplatin and PRP-treated group in comparison with the control group, but it is still less than the cisplatin-treated group.

## Discussion

Drug-induced nephrotoxicity is one of the leading causes of acute kidney injury (AKI). Many studies were conducted to study the effect of different drugs on the kidney. The most common drug to be used as a model was cisplatin because nephrotoxicity is one of its most common side effects [20, 21]. The aim of this work was to investigate the possible protective effect of PRP on the kidney in cisplatin-induced nephrotoxicity.

The dose of cisplatin used in the present work was 7.5 mg/kg, single dose. It was initially chosen according to a practice guide by Nair and Jacob [22]. Platelet-rich plasma (PRP) was prepared from the blood of five adult male rats. Male rats were chosen because of the theory adopted by Weil-Fugazza et al. [23]. which stated that the platelet count and growth factors in PRP increase during aging in male rats and slightly decrease in female rats.

In the current study, biochemical study of cisplatin-treated group showed significant increase in the level of plasma urea and creatinine; this could be explained by the destructive effect of cisplatin on the renal cells. Cisplatin decreases anti-apoptotic proteins and increases pro-apoptotic proteins such as p53 which causes renal cell apoptosis via activation of caspase 2, 3, and 8. Cisplatin also activates tumor necrosis factor (TNF) which leads to tubular necrosis. As a result, the renal functions are affected with impairment of urea and creatinine excretion and elevation of their serum level [24].

A study done by Sadeghi et al. [25] and El-Gizawy et al. [26] revealed renal damage after cisplatin treatment with increase in the serum level of urea and creatinine which was in agreement with the results of our work.

PRP significantly improved the renal functions. Biochemical study showed no significant difference in plasma urea and creatinine levels between control group and cisplatin and PRP-treated group. PRP in the present work was used as a blood product rich of growth factors in a trial to enhance renal tissue and function after administration of cisplatin [27, 28].

Findings of a study done by Keshk and Zahran [29] revealed that there was no significant difference between the control group and PRP-treated group regarding the renal functions in addition to restoration of the renal architecture. These results were similar to our study results.

With H&E stain in the present study, the kidneys of cisplatin-treated group showed severe degenerative changes such as necrosis of the lining epithelial cells of the proximal tubules, tubular cell vaculation, dilatation of the distal tubules, hyaline casts within the tubular lumina, inflammatory cellular infiltration within the renal interstitium, atrophy of the glomerular capillary tuft, and dilatation of the Bowman’s space.

The explanation of these degenerative changes is that cisplatin increases lipid peroxidation and malondialdehyde (MDA) through induction of oxidative stress. MDA induces histopathological changes in the kidney [30].

Inflammatory cellular infiltration can be explained by stimulation of pro-inflammatory molecules like TNF-α by cisplatin. These pro-inflammatory molecules play a great role in infiltration of macrophages and neutrophils within the kidney. Also T cells especially CD4 cells and, to a lesser degree, CD8 infiltrate within the kidney after cisplatin treatment. They also stimulate macrophages and other inflammatory cells to infiltrate the kidney [31].

Cisplatin leads to stimulation of reactive oxygen species which leads to oxidative stress and renal tubular injury. These injuries occur mainly within the proximal tubules because cellular uptake of cisplatin occurs through organic cation transporter 2 (OCT2), copper transporter 1 (CTR1) which are expressed mainly in the proximal convoluted tubules. This explains all degenerative changes within the proximal tubules such as loss of brush border and vaculations.

Tubular dilatation especially in the medullary rays and the distal convoluted tubules can be explained by the vascular injury caused by cisplatin. Cisplatin causes vasoconstriction through activation of adenosine A1 receptors (AT1s). It also causes damage to the vasculature within the renal tubules. This leads to vasoconstriction of vascular smooth muscle cells (VSMC) leading to reduced renal blood flow. This leads to increase intratubular pressure and dilatation of the renal tubules [32].

Findings of study done by Abd El-Rhman et al. [31] and Bazmandegan et al. [32] were in total agreement with the findings of the present work and stated that cisplatin causes severe degenerative changes within the cortex and medulla of the kidney such as coagulative necrosis of the tubular epithelial cells and eosinophilic casts within the tubular lumina.

Findings of a study done by Al Za’abi et al. [33] and Tripathi and Alshahrani [34] revealed severe necrosis of the lining cells of the proximal tubules, hyaline casts, and dilatation of the Bowman’s space which were in parallel with the findings of the present work.

With H&E stain in the present work, the kidneys of cisplatin and PRP-treated group showed more or less restoration of its normal structure with amelioration of the histological structure of the renal tubules, restoration of the normal glomerular structure, and the morphometric parameters were nearly similar to the control group. Only some inflammatory infiltration persisted in the sections of these groups as a trial for regeneration and restoration of the normal tissues.

This could be explained by the fact that PRP is considered as a cocktail of different cytokines and growth factors from the alpha granules present in the platelets. The level of growth factors in PRP is about 8-fold as compared to the whole blood [35].

The findings of study done by Salem et al. [36] stated that PRP accelerated recovery of renal functions in cisplatin nephrotoxicity; thus, it was proved to be a protective against cisplatin nephrotoxicity. These findings were aligned with the findings in this work.

With Masson’s trichrome stain, the cisplatin-treated group showed severe renal interstitial fibrosis. There was intense deposition of collagen fibers within both the medullary and cortical interstitium.

Renal interstitial fibrosis after cisplatin intake can be explained by different theories. The most popular and accepted one is that fibrosis is the endpoint of chronic kidney disease. Doses of about 7–9mg/kg can induce chronic kidney disease in the rats. In chronic kidney disease, there are chronic inflammatory cellular infiltration, chronic autophagy, and chronic endoplasmic reticulum stress. This leads to activation of myofibroblast cells with extracellular matrix deposition and interstitial fibrosis as the end result [37, 38].

Cisplatin-induced renal interstitial fibrosis can be also explained by epithelial mesenchymal transition in which the epithelial cells lose their cell polarity and cell-cell adhesion, and gain migratory and invasive properties to become mesenchymal stem cells [38].

A study done by Liang et al. [39] and Nakagawa et al. [40] showed that cisplatin caused intense renal interstitial fibrosis which was parallel with the findings in the present work.

Cisplatin and PRP-treated group showed marked decrease in the area of renal interstitial fibrosis when compared with cisplatin-treated group. Its score of the percentage of fibrosis was 1, whereas the cisplatin-treated group was score 3.

PRP decreases renal interstitial fibrosis through its anti-inflammatory effect. PRP increases the intracellular expression of anti-inflammatory mediators such as IL-4, IL-10, and IL-13 and suppress translocation of TNFa [41, 42].

Hegab et al. [43] in their study were in agreement with the results of the current work and highlighted the great role of PRP as anti-fibrotic agent.

With PAS stain in the present work, the cisplatin-treated group showed thickened and disrupted glomerular basement membrane. Also, there were severe tubular degenerative changes especially the proximal convoluted tubules which appeared with thickened tubular basement membrane and disrupted apical brush border.

Thickening of the glomerular basement membrane can be explained by cisplatin-induced oxidative stress. It causes DNA damage by forming DNA adducts. Thus, it leads to cell cycle arrest and cell apoptosis. One of the affected cells is podocyte which has a central role in maintaining normal glomerular basement membrane structure. Injured podocyte leads to upsetting of the balance between basement membrane’s synthetic and degradative pathways [44, 45].

Meprins are oligomeric metallo-proteinases located normally in the brush border of the proximal convoluted tubules. They are able to cleave a basement membrane protein called nidogen-1. Cisplatin leads to alteration of the position of meprins from the apical membrane of the proximal convoluted tubules towards the basolateral surface which leads to cleavage of nidogen-1 protein and this explains disruption of both tubular and glomerular basement membrane by cisplatin [46].

Rachid et al. [47] reported in their study that cisplatin led to increase the thickness of the glomerular basement membrane in addition to disruption of the brush border of the proximal convoluted tubules. These data were in a close correlation with the data in the present work.

Cisplatin and PRP-treated group showed slight increase in the thickness of the glomerular basement membrane. It showed restoration of the tubular structure especially the proximal convoluted tubules which appeared with nearly normal basement membrane and intact brush border.

Cisplatin and PRP-treated group showed restoration of the normal tubular and glomerular structure because of the anti-apoptotic properties of PRP. PRP downregulates the expression of apoptotic genes as DAPK1 and BIM mRNA in addition to inhibition of P53, BAX, and caspase-3 [36].

PRP releases growth factors (GFs) such as hepatocyte growth factor (HGF), adenosine diphosphate (ADP), adenosine triphosphate (ATP), epidermal growth factor (EGF), and insulin-like growth factor-1 (IGF-1). These growth factors especially IGF-1 enhance tubular cell regeneration [36].

Immunohistochemically, the cisplatin-treated group showed strong positive anti caspase-3 reaction in many cells of the renal tubules especially the ballooned tubules with attenuated cells in addition to the interstitium. There were multiple areas of tubular apoptosis in addition to apoptotic cells which appeared as brownish areas.

These results can be explained by a theory adopted by Al Za’abi et al. [33] and Panahi et al. [48] who suggested that cisplatin accumulate in the renal tissue during glomerular filtration and tubular secretion. It induces activation of reactive oxygen species and the cascade of cell death signaling. This leads to apoptosis and necrosis of the renal cells [33, 48].

A study done by Shalan and Abd El Fattah [49] showed that cisplatin caused severe caspase-3 activity in the renal tissue especially the renal tubules which was parallel with the findings in the present work.

Cisplatin and PRP-treated group showed significant decrease in the caspase-3 activity in apoptotic cells in comparison with the cisplatin-treated group. There were few focal areas of tubular cell apoptosis. This can be explained by the anti-apoptotic activities of PRP. PRP is known to inhibit pro-apoptotic mediators such as caspase-3 and P53.

Morphometric analysis showed extensive morphological damage in the cisplatin-treated group in comparison with the control group. Cisplatin-treated group had enlarged glomeruli in comparison with control group. This was confirmed by the high values of renal corpuscular perimeter and Feret’s diameter. There was no difference between this group and the control group according to the glomerular circularity.

The cisplatin-treated group showed higher values of the glomerular basement membrane thickness in comparison with the control group. The Bowman’s space showed severe dilatation in comparison with the control group. This was confirmed by the high percentage of the surface area of Bowman’s space in relation to the surface area of the corpuscle.

Cisplatin-treated group showed severe tubular damage in the form of luminal dilatation, vaculation, cell swelling and degeneration, and accumulation of hyaline casts within their lumens. This was confirmed by the high percentage of abnormal tubules in relation to the normal tubules within this group.

Tubular toxicity especially proximal tubule toxicity is due to accumulation of cisplatin and its toxic metabolites in the renal tubules especially the proximal tubules [13].

Study done by Ilić et al. [50] showed enlarged renal corpuscles which were confirmed by the high values of glomerular perimeter and Feret’s diameter. These results were in parallel with the morphometric findings revealed in the present work.

Morphometric analysis showed that there was nearly restoration of the normal renal architecture in cisplatin and PRP-treated group. There was improvement in the morphometric parameters in comparison with the cisplatin-treated group. The glomeruli were smaller in size in comparison with the cisplatin-treated group. This was confirmed by the lower values of glomerular perimeter and Feret’s diameter.

The basement membrane was thinner than the cisplatin-treated group. The percentage of abnormal tubules in relation to the normal tubules was lower within this group. The Bowman’s space showed lesser dilatation in comparison with the cisplatin-treated group. This was confirmed by the lower percentage of the surface area of Bowman’s space in relation to the surface area of the corpuscle.

This could be explained by the Reno protective properties of PRP. PRP is known to have great concentrations of growth factors. These growth factors especially IGF-1 enhance tubular cell regeneration. PRP has anti-apoptotic properties through downregulation of the expression of apoptotic genes as DAPK1 and BIM mRNA [35, 36].

In agreement with the findings of the present work, a study done by Salem et al. [36] stated that PRP accelerated recovery of renal functions in cisplatin nephrotoxicity and restored of the normal renal structure as regard the renal glomeruli and tubules.

In the current work, we concluded that PRP has a protective effect on renal tissues and it ameliorated the normal histological structure and function of the renal tissue after cisplatin-induced nephrotoxicity.

## Conclusions

Cisplatin cause destruction in the renal tissues in addition to deterioration of the renal functions. PRP ameliorated these histological changes**.** Concomitant use of PRP with cisplatin helps to preserve the renal tissue structure and function and mitigate the nephrotoxic effects of cisplatin.

### Limitations of the Study

This study has small sample size.

## Data Availability

The datasets used and/or analyzed during the current study are available from the corresponding author on reasonable request.
